# Analysis of dataset on editorial board composition of Dove Medical Press by continent

**DOI:** 10.1016/j.dib.2018.08.196

**Published:** 2018-09-05

**Authors:** Hilary I. Okagbue, Aderemi A. Atayero, Muminu O. Adamu, Pelumi E. Oguntunde, Abiodun A. Opanuga, Patience I. Adamu

**Affiliations:** a Department of Mathematics, Covenant University, Canaanland, Ota, Nigeria; b Department of Electrical and Information Engineering, Covenant University, Canaanland, Ota, Nigeria; c Department of Mathematics, University of Lagos, Akoka, Lagos, Nigeria

**Keywords:** Dove Medical Press, Bibliometrics, Data analysis, Random, Smart campus, Ranking analytics, Statistics

## Abstract

This article presents the frequency of distribution of editorial members of Dove Medical press, across the world based on their official stated affiliations. Uneven distributions across the six continents were observed and this was confirmed by the Chi-square test of goodness of fit. Further research can focus on data on the gender composition, distribution of the affiliations of the first or corresponding authors of the respective journals, citation and editorial board composition based on the abstraction and indexation of the journals.

**Specifications table**

**Table:** 

Subject area	Decision Sciences
More specific subject area	Bibliometrics, Statistical data analysis
Type of data	Table, Figure and MS Excel
How data was acquired	The data was obtained from freely open access Dove medical press journals which is a part of Taylor and Francis Group, the academic publishing arm of Informa PLC
Data format	Raw, partially analyzed
Experimental factors	Patterns of distribution of editorial members across the world based on their official stated affiliations.
Experimental features	Statistical analysis of editorial composition across the continents
Data source location	Dove Medical Press
Data accessibility	All the data are in this data article

**Value of the data**

• The dataset can be helpful in bibliometric and research evaluation analysis.
• The data analysis can be extended to obtain the editorial board composition based on the journal indexing such as Scopus, PubMed, Web of Science and so on.
• The dataset can provide insight to the theoretical and observed expectation of the editorial board composition using the Chi-square test of goodness of fit.
• The data can be helpful in efficient smart campus and journal management.
• The data analysis can be extended to include the gender composition and distribution of the affiliations of the corresponding or first authors of the journals.
• Other known statistical tools may be applied to the dataset for more exploration.

## 1 Data

The dataset contained in this article are the official stated affiliations of the members of the editorial boards of 109 journals published by the Dove medical press. The editorial board membership of each journal comprises of the editor in chief, associate editors, statistical editor (if applicable) and the other members of the editorial board. The affiliations are grouped according to the six continents. The continents are denoted as follows: North America (NAM), Europe (EURO), Asia (ASIA), South America (SAM), Australia (AUST) and Africa (AFR). The raw dataset can be assessed as Supplementary data. Similar dataset can be obtained which can focus on data on the gender composition, distribution of the affiliations of the first or corresponding authors of the respective journals, citation and editorial board composition based on the abstraction and indexation of the journals such as Emerging Sources Citation [1], Scopus [2] and PubMed [3].

### 1.1 The total editorial board composition

The description statistics was obtained for the total editorial board composition of the 109 journals. This is shown in Table 1.

Remarks As of this writing, Dove Medical press has a total of 2120 editorial board members for 109 journals. The dataset is moderately positively skewed with a coefficient of skewness of 1.317 (this is because of the presence of a few extreme values). On the average, any randomly selected journals would have approximately 19±8 people in its editorial board.

**Table 1:** Descriptive statistics of the total editorial board composition of 109 Dove medical press.

N		190
Mean		19.45
Median		17
Mode		16
Std. Deviation		8.18
Variance		66.916
Skewness		1.317
Std. Error of Skewness		0.231
Kurtosis		1.946
Std. Error of Kurtosis		0.459
Range		42
Minimum		8
Maximum		50
Sum		2120
Percentiles	10	11
	20	13
	25	14
	30	15
	40	16
	50	17
	60	19
	70	22
	75	23.5
	80	25
	90	32
	95	36

### 1.2 Distribution of editorial board membership across the six continents

**Fig. 1:**
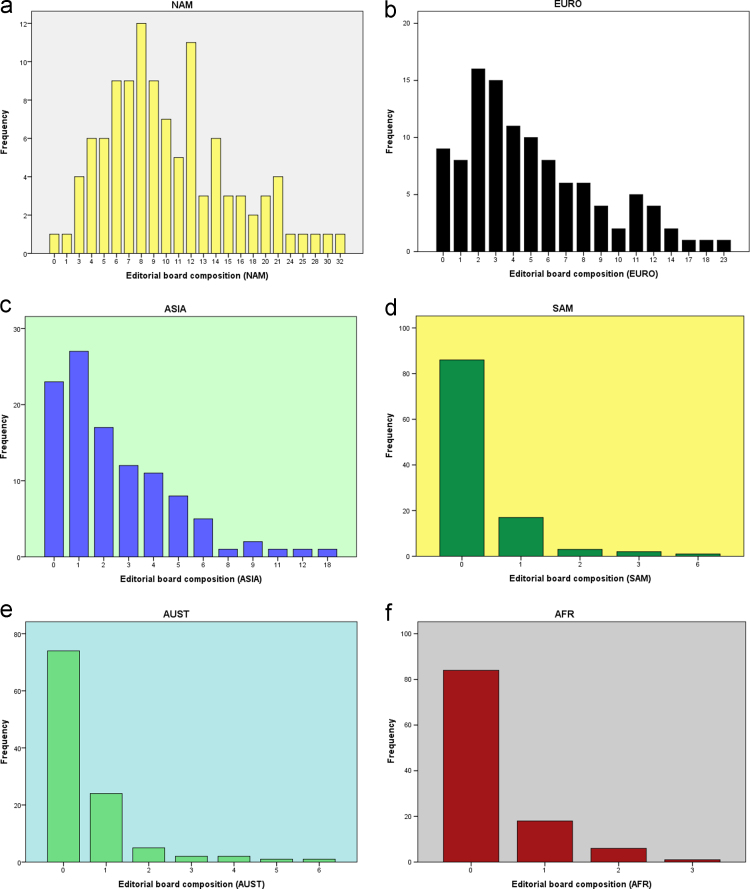
**a**: The frequency of the editorial board composition (North America). **b**: The frequency of the editorial board composition (Europe). **c**: The frequency of the editorial board composition (Asia). **d**: The frequency of the editorial board composition (South America). **e**: The frequency of the editorial board composition (Australia). **f**: The frequency of the editorial board composition (Africa).

Descriptive statistics of the respective six continents are done to explore the patterns of distribution that is only summarized in Table 1. This is shown in Table 2. The frequency is shown in details in Fig. 1**a, b, c, d, e** and **f**.

Remarks The percentage of the total sum across the continents is as follows: North America (54%), Europe (26.5%), Asia (13%), South America (1.65%), Australia (2.78%) and Africa (1.56%).

**Table 2:** Descriptive statistics of the editorial board composition of 109 Dove medical press across the six continents.

		NAM	EURO	ASIA	SAM	AUST	AFR
Mean		10.58	5.16	2.55	0.32	0.54	0.30
Std. Error of Mean		0.574	0.406	0.271	0.078	0.102	0.059
Median		9.00	4.00	2.00	0.00	0.00	0.00
Mode		8	2	1	0	0	0
Std. Deviation		5.997	4.239	2.830	0.815	1.067	0.616
Variance		35.968	17.966	8.009	0.664	1.139	0.380
Skewness		1.255	1.397	2.374	4.149	2.868	2.126
Kurtosis		1.905	2.623	8.598	22.886	9.523	4.225
Range		32	23	18	6	6	3
Minimum		0	0	0	0	0	0
Maximum		32	23	18	6	6	3
Sum		1153	562	278	35	59	33
							
Percentiles	10	4.00	1.00	0.00	0.00	0.00	0.00
	20	6.00	2.00	0.00	0.00	0.00	0.00
	25	6.50	2.00	1.00	0.00	0.00	0.00
	30	7.00	2.00	1.00	0.00	0.00	0.00
	40	8.00	3.00	1.00	0.00	0.00	0.00
	50	9.00	4.00	2.00	0.00	0.00	0.00
	60	11.00	5.00	2.00	0.00	0.00	0.00
	70	12.00	6.00	3.00	0.00	1.00	0.00
	75	13.00	7.00	4.00	0.00	1.00	0.00
	80	14.00	8.00	4.00	1.00	1.00	1.00
	90	20.00	11.00	6.00	1.00	2.00	1.00
	95	22.50	13.00	8.50	2.00	3.00	2.00

## 2 Experimental design, materials and methods

The data were manually extracted from the publisher׳s websites and analyzed using SPSS 20.0 and Microsoft Excel. The data were current as at the time of writing this article and a complete enumeration of the board members of Dove press. It was also observed that some researchers are board members in more than one journal and that necessitated that the reason why each journal was treated individually from the web page related to it. The board members are classified according to six continents. Journals that are no longer accepting papers for publications were not considered. The design adopted for data collection is because of the importance of the editorial board members in journal management, which includes; paper submission, appointment or recruitment of reviewers, decisions on submissions made by authors, quality assurance, indexation and abstraction, disciplinary roles in cases of scholarly misconduct and so on.

Similar works can be considered, see [4], [5], [6], [7], [8], [9], [10], [11], [12], [13], [14], [15], [16], [17], [18], [19], [20], [21], [22], [23], [24], [25].

The data is characterized by an uneven editorial board distribution across the continents. This can be investigated by the use of Chi-square test of goodness of fit test. The assumption of no underlying probability was the reason of the use of Chi-square test of goodness of fit. This is summarized in Table 3.

Remarks The distribution of the editorial board across the continents is highly uneven as evidenced by the p-values. The implications of this as regards to gender equality, acceptance and rejection rates of manuscripts and rate of publications are subject to further investigations.

**Table 3:** Chi-square test of goodness of fit for the data across the continents.

Test	NAM	EURO	ASIA	SAM	AUST	AFR
Chi-Square value	60.321	54.917	101.165	244.165	281.908	163.183
Degrees of freedom	23	16	11	4	6	3
p value	0.000	0.000	0.000	0.000	0.000	0.000
